# HPV Self-Sampling Promotion Among African American (AA) and Sub-Saharan African (SAI) Immigrant Women: Adaptation and Usability Testing

**DOI:** 10.3390/ijerph22030317

**Published:** 2025-02-20

**Authors:** Adebola Adegboyega, Gia Mudd-Martin, Nancy E. Schoenberg, Mark Dignan

**Affiliations:** 1 College of Nursing, University of Kentucky, Lexington, KY 40536-0232, USA; gia.mudd@uky.edu; 2 Department of Behavioral Science, College of Medicine, University of Kentucky, Lexington, KY 40536-0298, USA; nesch@uky.edu; 3 Department of Internal Medicine, College of Medicine, University of Kentucky, Lexington, KY 40536-0298, USA; mark.dignan@uky.edu

**Keywords:** cervical cancer, screening, human papillomavirus self-sampling, education, African American, sub-Saharan African immigrant

## Abstract

Background: Cervical cancer (CC) rates have declined nationally but rates remain high in Black women with most cases occurring among unscreened and under-screened women. This paper describes the adaptation, acceptability, and useability of an education intervention, “Health is Wealth: A Cervical Health Intervention”, to promote cervical screening and reduce perceived barriers to CC screening among two subgroups of Black women: African American (AA) and sub-Saharan African immigrant (SAI) women. Methods: In this paper, we describe the process of adapting the Health is Wealth intervention using the Barrera and Castro adaptation framework. The iterative adaptation process included formative focus groups (n = 30 participants) to gather information, expert feedback (n = 4), and usability testing (n = 7). Results: The systematic process resulted in the modification of educational intervention components. Several aspects of the intervention were modified, and core elements of the original intervention were preserved. The usability testing findings suggest the intervention would support the objective of promoting cervical cancer screening uptake among AA and SAI women. Conclusions: Adaptation of an evidence-based intervention is necessary to ensure contextually and culturally appropriateness for target populations, particularly for minoritized populations. We demonstrated that an evidence-based intervention for Pap screening can be adapted for HPV-self-sampling promotion with target community input.

## 1. Introduction

In the U.S., Black women experience higher cervical cancer incidence and mortality than White women [1]. Cervical cancer incidence among Black women (7.3 per 100,000 persons) is one of the highest of any minorized population and mortality rates (3.2 per 100,000 persons) are significantly higher than their non-Black counterparts including Native Americans, Asians, Hispanics, and Whites (1.9, 1.7, 2.5, and 2.1 per 100,000 persons, respectively) [2]. The overall 5-year relative cervical cancer survival rate among Black women is 58%, compared to 65% for Native Americans, 67% for White, and 72% for Hispanic women [3].

Cervical cancer screening continues to be a key factor in secondary prevention for unvaccinated and HPV-vaccinated women. Being up to date with recommended screening and HPV vaccination can dramatically reduce cervical cancer. The U.S. Preventive Services Task Force and other organizations recommend clinic-based HPV tests and/or co-testing (HPV test in combination with cytology testing) for women aged 30 to 65 years. Most cases of new cervical cancers are in women who have never been screened or have not been screened in the previous 5 years of their lives [4]. Despite similar screening rates for Black and White women (74.8 vs. 75.4) [2], both groups fall short of the Healthy People 2030 target to increase screening to 84.3 percent among women aged 21 to 65 years who receive cervical cancer screening [5].

U.S.-born Black women, or African Americans (AAs), and Black women who are immigrants are often grouped together in health research which may skew the data leading to distortions that may mask differences in important determinants of health including beliefs, behaviors, risk factors, disease experiences, culture, and health status [6,7,8,9]. Cancer screening disparities have been documented for immigrants with immigrants being less likely to receive a Pap test even when compared to the uninsured U.S.-born Black population [10,11,12,13]. In one study, data from the National Health Interview Survey from 2005 to 2015 were pooled to examine differences in Pap test receipt among women by birthplace and percent of lifetime in the U.S. The results showed that foreign-born women were more than twice as likely as U.S.-born women to have never had Pap testing and that sub-Saharan African immigrant (SAI) women were more than four times as likely as U.S.-born women to have never received a Pap test (27.8% vs. 6.8%, respectively) [10]. Differences in Pap test receipt also existed based on the number of years in the U.S. Women who had spent less than 25% of their lifetime in the U.S. were less likely to have been screened compared to foreign-born women who spent more than 25% of their lives in the U.S. (12.7%) [10].

There are several barriers to cervical cancer screening uptake. Patient-level barriers include lack of optimal self-care and use of preventive services, low socioeconomic status, insufficient knowledge, negative healthcare experiences, fear of tests, logistical barriers, poor follow-up of abnormal results, modesty, length of residence in the U.S., and fatalistic beliefs [11,12,13]. Among common provider-level factors are discrimination, failure to provide age- and risk-appropriate recommendations, and poor patient–provider communication. Among the most prevalent healthcare barriers are low-quality services, health insurance factors, and issues that limit access to care [14,15,16]. Research that can inform effective approaches to overcome these barriers and increase screening among women who are underserved and under-screened is critical to developing interventions that will lead to improved screening.

HPV self-sampling represents one strategy that may promote women’s engagement in screening by overcoming barriers to completing clinic-based cervical cancer screening [8,15]. Self-sampling provides an effective approach for the early detection of cervical cancer for women aged ≥ 30 years that does not require an office visit. While samples for HPV testing are typically collected by a healthcare provider in a clinical setting, research shows that women prefer to collect their own samples for HPV testing because of ease of use, convenience, privacy, and decreased embarrassment [15]. HPV self-sampling is a newer screening modality that is well validated with comparable sensitivity and specificity to physician-collected samples for detecting high-grade cervical lesions [16,17]. Despite these benefits and high acceptability of HPV self-sampling by vulnerable and underrepresented populations, there is low self-efficacy related to the use of sampling kits, with women expressing concern that they do not have the skills to appropriately collect samples, fearing self-harm, and apprehension that self-sampling will replace gynecological visits [10,14,15,16,17].

The concerns identified by women related to HPV self-sampling can be addressed through tailored education to support overcoming perceived barriers to self-sampling and cervical cancer prevention. Some educational interventions have demonstrated promising results in increasing cervical cancer screening by reducing barriers women experience [18,19,20], particularly if they address the specific needs of a target population through the use of strategies tailored for that community [21]. While there have been positive outcomes of education intervention to promote HPV self-sampling in several populations of women with low screening rates, there is limited literature that details the adaptation and useability of education interventions for AA and SAI women. The purpose of this paper is to describe the adaptation of a single-session education intervention and to test the acceptability and usability of an adapted cervical health education intervention to two subgroups of Black women, AA and SAI.

### Theoretical Framework

Because an individual’s health is influenced by multiple factors in the physical and social environment in addition to personal attributes and behaviors, we draw on both the Social Cognitive Theory [22] and the Health Belief Model [23] as theoretical underpinnings for this study. The Social Cognitive Theory posits that behavior is the product of the dynamic interplay of personal, behavioral, and environmental influences. The Health Belief Model suggests that a person’s beliefs, attitudes, and perceptions about a disease determine their actions to seek methods to prevent, screen for, and control that disease. For the purpose of this study, the education content was grounded in the Health Belief Model and Social Cognitive Theory with a focus on influencing personal behavioral constructs such as prerequisite knowledge and skills to overcome barriers to screening and promote screening engagement.

## 2. Materials and Methods

### 2.1. Study Setting and Recruitment

This community-based study was designed to engage AA and SAI women in the process of adaptation and development of a cervical health intervention. The study took place in Kentucky with participants recruited from community locations sites in Fayette, Jefferson, and Franklin counties. The University of Kentucky Office of Research Integrity (IRB # 60704) approved the research. All methods were conducted in accordance with relevant guidelines and regulations, and no research procedures commenced prior to University Institutional Review Board approval.

### 2.2. Intervention Adaptation Process

#### 2.2.1. Overview of the Intervention to Be Adapted

The Gateway to Health: A Cervical Cancer Screening program [24], heretofore referred to as the Gateway to Health program, was selected for adaptation because it is an evidence-based cancer control program that successfully increased cervical cancer screening among Korean Americans [24]. Given that this theoretically grounded education intervention was developed for a minoritized population with culturally appropriate adaptations, there was potential for adaptation for AA and SAI women. Detailed information about the program is reported on the National Cancer Institute, Evidence-based Cancer Control Programs website [25].

The first author consulted the program developer prior to the adaptation of the intervention. The Gateway to Health program was developed for awareness building and behavior modification for Korean American women ages 21 and older to promote cervical cancer screening with Pap screening uptake [24]. The intervention group received a single 2 h educational session that focused on cervical cancer risk factors, screening guidelines, and procedures, a discussion of possible barriers to screening relevant to Korean American women, with navigation services conducted by bilingual community health educators. Participants in the control group also received a one-time 2 h education session focused on general health and cancer education, nutrition, benefits of obtaining routine medical checkups, and descriptions of cancer screening tests along with recommended screening guidelines. In the efficacy trial, 347 Korean American women were enrolled in the intervention compared with the control (n = 358). The intervention led to significantly higher screening rates (odds ratio [OR], 25.9; 95% confidence interval [CI], 10.1–66.1, *p* < 0.001). Intervention participants showed improved knowledge and self-efficacy for cervical cancer screenings.

#### 2.2.2. Adaptation Process and Outcomes

There was a sequence of four intervention adaptation stages: (a) information gathering, (b) preliminary adaptation design, (c) preliminary adaptation testing, and (d) adaptation refinement based on recommendation by Barrera and Castro [26]. The goal of the information gathering stage is to identify ideas to become better informed about the form, content of needed adaptation, and preferences of potential participants. During the preliminary adaptation design, information gathered in the first stage is developed into draft materials, and additional ideas are gathered from potential participants. In the preliminary adaptation test phase, investigators conduct pilot studies with small groups to determine if the desired goals of adaptation were achieved and to identify and discuss sources of program nonfit, implementation difficulties, or difficulties with program content or activities. Adaptation refinements focus on using experience with pilot studies to inform a revision of intervention procedures [26].

**Figure 1:**
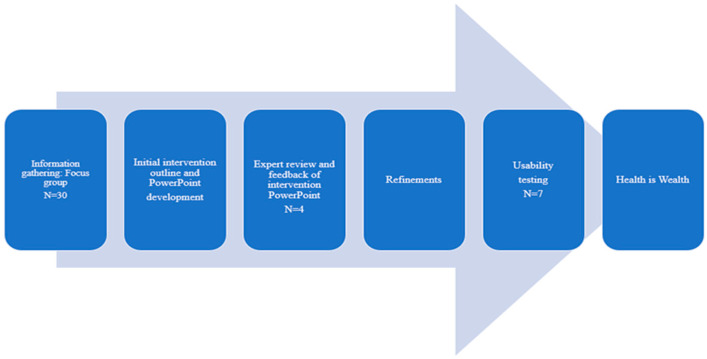
Iterative process for developing Health is Wealth intervention.

To gather information, thirty Black (AA and SAI) women participated in one of six focus groups conducted to inform the adaptation of the intervention. Following adaptation, four cancer control researchers provided an expert review of the preliminary intervention contents and procedures. The feedback informed additional refinements to the intervention PowerPoint. We then conducted a usability test with seven women (AA, n = 4; and SAI, n = 3). The literature supports that a small sample (n ≤ 10) may suffice for assessing clarity of instructions or item wording, acceptability of formatting, or ease of administration of an intervention [27]. Thus, this sample size was sufficient to uncover issues with the intervention, determine the acceptability of the intervention contents, and feasibility of protocol, and identify areas that can be enhanced prior to the intervention implementation. In addition, we collected qualitative feedback and reached saturation. The women were presented with the adapted intervention materials that resulted from the prior two processes and provided feedback. We used an iterative process, shown in Figure 1, to adapt and revise the intervention components. Members of the research team for this study have broad expertise in behavioral intervention development, cancer control and prevention, immigrant health, and experience with cultural, psychosocial, and environmental determinants of health disparities in underserved populations that was leveraged to systematically adapt the education intervention.

#### 2.2.3. Information Gathering and Preliminary Adaptation Design

As recommended by Barrera and Castro [26], to adapt the content of Gateway to Health for use for AA and SAI women, we conducted formative research with the target population. This informed changes to the educational components and to the handout to enhance cultural and contextual relevance for use with AA and SAI women. For the preliminary adaptation design stage, several aspects of the intervention were modified and core elements of the original intervention were preserved unless there was reason that a core element should be dropped [26] to meet cultural appropriateness and intervention objectives.

### 2.3. Focus Groups

Using purposive sampling [28], we recruited thirty AA and SAI women from community settings including churches, community centers, and beauty salons using study-approved flyers, social media, and word of mouth to participate in focus groups. Women aged 18–65 years who were English-speaking, self-identified as AA or SAI, and who lived in Kentucky were eligible to participate. Participants were offered a $15 gift card as an incentive for their time. Six focus groups were conducted at which point we reached thematic saturation [29]. Each focus group was facilitated by a Black woman trained in conducting qualitative interviews. The interviewer started each focus-group session with an introduction by describing the purpose of the study, the objectives of the focus group, and how the results from the interview sessions will be used and disseminated. The facilitator provided information about HPV testing for cervical cancer screening, and the rationale for conducting HPV testing using self-collected samples. Each focus-group session was guided with a semi-structured interview guide and additional probes. Focus group questions were related to concerns that prevent women from using an HPV self-sampling kit, important considerations to promote uptake, follow-up with women who do not return a kit, and provision of results to women following an HPV self-sampling completion. Through the focus-group sessions, we were also able to identify components that needed to be addressed to improve the cultural acceptability of the cervical health intervention.

### 2.4. Analysis

Focus-group sessions were audio-recorded and transcribed for analysis. Transcripts were corrected as needed when any transcription error was detected. Data were analyzed using qualitative content analysis to identify themes that emerged. We used Microsoft Word™ and an Excel™ spreadsheet to organize the data [30]. Two researchers with expertise in qualitative analyses read the transcripts to familiarize themselves with the data. The researchers used line-by-line coding to identify emergent themes. Line-by-line coding involves summarizing the data text into small categories of information and assigning a label to the code [29]. The two researchers met to compare identified themes. Differences in coding and themes were resolved through discussion. Representative quotes were jointly selected that supported each theme.

## 3. Results

### 3.1. Focus-Group Findings

The focus-group sample included AA (n = 16) and SAI (n = 14) women whose mean age was 33.67 ± 9.03 years. The majority were not currently married (53.3%) and did not have health insurance (53.3%). Three themes, including empowerment, comprehensive cervical health education, and confidentiality, emerged from the focus group discussions. The themes, concepts, and exemplary quotes are summarized in Table 1.

### 3.2. Initial Outline and PowerPoint Slides

Using the outline from Gateway to Health, an initial outline and PowerPoint for a cervical health intervention was developed by the first author. The education consisted of eight components that included an overview of cervical cancer, data on cervical cancer for AA and SAI women, cervical cancer risk factors, prevention strategies, screening guidelines, common myths about cervical cancer and HPV testing, barriers to screening, information about the availability of HPV self-sampling for screening, the procedure for completing HPV self-sampling, and community resources to promote screening. We incorporated credible information from lead health organizations, e.g., National Institute of Health, American Cancer Society, World Health Organization, and Centers for Disease Control and Prevention.

Focus-group findings were used to modify and refine intervention content, PowerPoint slides, and the protocol for implementation. The findings informed the adaptations for AA and SAI. For example, focus-group participants suggested that women be shown an example of how to perform a self-sampling. In response, an HPV self-sampling tutorial video was included as part of the PowerPoint presentation, in addition to the step-by-step information included in the self-sampling kit from the manufacturer.

While there are broad cultural similarities among Black women, there are inter- and intra-group differences in health beliefs, migration history, and literacy that presumably might affect participation in cervical cancer screening [7,31,32]. SAI women’s cultural beliefs of diseases, language discordance, religiosity, and spirituality inform the ways they perceive their health and healthcare experiences. For example, research suggests that SAI women are reluctant to go for cancer screening, because of their belief that their health is determined by God [33], while some believe they are not vulnerable to cancer because of their prayers and faith [17].

The investigators adapted the intervention materials for use with AA and SAI women by addressing health beliefs and language barriers and prioritizing values relevant to AA and SAI women. The content was written at a fifth grade reading level for both groups and short, focused messages were used. For the educational session, two case studies were developed to facilitate discussion, one for AA women and the other for SAI women, responsive to each group’s unique experiences.

### 3.3. Preliminary Adaptation Test and Refinement Phase

For the preliminary adaptation test and refinement phases we received input and feedback from three cancer control and prevention experts and one behavioral researcher. Among the four experts, one had a doctoral degree in a relevant discipline, two had expertise in education intervention and cancer screening research, another had expertise in community-engaged research and use of HPV self-sampling, and one had expertise in behavioral intervention research with minoritized populations. The first author knew the researchers prior to the study commencement, and each was contacted via email to serve as an expert. After each agreed, the first author shared the purpose of the study and requested expert feedback on the intervention outline and PowerPoint slides. The experts reviewed the PowerPoint and provided critiques and recommendations for improvements via written comments on the PowerPoint slide deck and during a one-on-one Zoom meeting with the first author. Overall, feedback focused on the informational content of the PowerPoint and the clarity of the content; it was also suggested to develop a script to guide the PowerPoint presentation for intervention fidelity. Focus-group findings and expert review suggestions and feedback were integrated into the adaptation and informed further refinements to the intervention.

#### 3.3.1. Peer Educators Training

In preparation for conducting a preliminary assessment of the adapted intervention, two peer educators with good communication skills and comfort working with the communities of focus were recruited, one from the AA community and one from the SAI community. Peer educators are trusted and trained laypeople who are familiar with the community to which they provide education and who have similar backgrounds and characteristics as the target population [34]. The peer educators completed training in human subject protection. Members of the research team provided training in the data collection protocol. The team also created an intervention script to standardize the delivery of the Health is Wealth intervention. The peer educators were trained in how to deliver the intervention. They also completed intervention fidelity certification prior to conducting a session.

#### 3.3.2. Usability Testing

Women who self-identified as being AA or SAI were English-speaking, and who had a cell phone or other device to attend a Zoom session were eligible to participate. Participants were recruited using study-approved flyers and word-of-mouth to participate in a usability test in order to determine the acceptability of the intervention contents and feasibility of protocol and to gather final feedback for refinement prior to the main trial.

Seven women (AA, n = 4 and SAI, n = 3) consented to participate in the study. They received an electronic version of the educational PowerPoint via email ahead of the session. The peer educators scheduled a 1 h educational session with participants. The sessions were held via Zoom. The intervention content was delivered by an SAI peer educator for the SAI group and an AA peer educator for the AA group.

The presenter provided the education using a Health is Wealth PowerPoint slides presentation during which the peer educator asked a series of questions related to the acceptability of the intervention content and pictures, as well as the clarity of the information. During the intervention delivery, the peer educator cultivated a supportive and conducive learning environment to promote participants’ engagement and encourage questions throughout. The participants completed a short survey following the session to assess the acceptability, relevance, and usefulness of the instruction. Survey items were rated on a response scale ranging from 1 (strongly disagree) to 5 (strongly agree). Participants provided general feedback after the session and in an open-ended item on the survey. Results from the feedback provided during the sessions and via the post-intervention survey were used to guide further refinement of Health is Wealth. Participants were offered a USD 30 gift card as an incentive for their time. Survey items were analyzed using descriptive statistics (i.e., means and standard deviation). Qualitative feedback was analyzed by methods similar to the focus group analysis described above.

### 3.4. Findings

Qualitative feedback showed satisfaction with the education intervention. One woman stated, “the information was well organized in its presentation and each component increased knowledge of the topic. Flowed well”. Another woman stated, “the program seeks to provide valuable information on the health issue raised”. Overall, the participants liked the option to attend the session virtually and enjoyed the opportunity to contribute to the research.

Feedback from usability testing is presented in Table 2. Feedback showed a high level of acceptability (mean = 4.7), that the intervention materials were relevant (mean (n = 4.5)), and that the HPV-sampling instruction was useful (mean (n = 4.2)). The participants expressed enthusiasm about the importance of the study and provided additional information for consideration and to guide participants’ recruitment. For example, participants suggested that more explanation should be included about how smoking and reproductive factors such as having many children could increase the risk of cervical cancer. In response to this feedback, we included more information to further explain modifiable and non-modifiable risk factors of cervical cancer. Using information from reputable organizations, we provided a detailed rationale of how smoking and reproductive factors can increase cervical cancer risk. Based on this feedback, additional refinements were made to the intervention prior to finalizing plans for implementation in a trial. In addition, participants suggested recruitment through social media advertisements, family resource centers, community and religious organizations, sororities, and radio advertisements.

## 4. Plans for Implementation and Evaluation of the Adapted Intervention: Health Is Wealth: A Cervical Health Program

The Health is Wealth intervention was adapted from the Gateway to Health program for the target population of Black (AA and SAI) women using the well-established adaptation framework by Barrera and Castro [26]. To ensure the balance between adhering to the original intervention (Gateway to Health) and cultural fit we maintained the core elements of the intervention and incorporated new elements to address cultural barriers and leverage identified motivators to increase cervical screening among AA and SAI women. Identifying risk and protective factors unique to a community and addressing these within an intervention have the potential to increase the efficacy of the intervention [35]. For Health is Wealth, the provision of an HPV self-sampling kit was added as a new component to the intervention to address access barriers to cervical cancer and empower women to complete cervical screening. We retained the use of trained trusted lay educators who understand cultural nuances and lived experiences within local communities to deliver group education to AA and SAI women. Due to the COVID-19 pandemic, we changed the in-person education session delivery to virtual delivery sessions over Zoom. In place of a reminder letter, we added a one-month follow-up call to have a personal connection with women and an opportunity to answer any question related to self-sampling or clinic-based cervical screening. Table 3 details the original and adapted components of the intervention.

Health is Wealth is a multicomponent intervention. The first component is a one-time 1 h education with seven components provided by a trained peer educator using a PowerPoint presentation format over Zoom. The education components include information on cervical cancer risk factors and prevention strategies and addressing common myths about cervical cancer and HPV testing. The education includes a skill-building activity using two case studies and a discussion on available low-cost or free screening programs. The second intervention component is the provision of an HPV self-sampling kit accompanied by instructions. Before the education session, participants are mailed or hand-delivered an HPV sampling kit accompanied by a manual that describes how to use the kit and return the completed test to the laboratory. The third intervention component is the provision of navigation for women who receive a positive HPV test result. Navigation includes guidance on the next steps, identifying local clinics that provide free screening and offer extended hours as well as referral to further screening. To assess the preliminary efficacy of Health is Wealth, a prospective one-arm quasi-experimental pilot trial among AA and SAI women aged 30 to 65 years who are out of compliance with cervical cancer screening is ongoing. The eligibility criteria for participants include women who (1) self-report not having a Pap smear within the past 3 years or Pap smear/HPV co-test within the past 5 years or HPV test within the past 5 years (per USPSTF guidelines); (2) are 30–65 years of age, as HPV testing is recommended for women in this age range per USPSTF guidelines; (3) are English speaking; (4) self-identify as an African American or SAI; and (5) who have a cell phone to participate in a virtual session. Exclusion criteria include current pregnancy, history of hysterectomy, and history of cervical cancer.

## 5. Discussion

This paper describes the iterative process of the adaptation and development of an evidence-based intervention. The adaptation and usability testing produced a cultural and contextual appropriate intervention, Health is Wealth: A Cervical Health Program. Program modifications are intended to increase the fit of the intervention to the target population while protecting scientific integrity [36]. For this adaptation, we modified the educational PowerPoint slides and protocols to resonate with AA and SAI women to improve satisfaction and acceptability. Adapting an existing evidence-based intervention has the potential to improve health equity with greater efficiency, effectiveness, and cost savings than initiating a program from scratch [36]. This paper fills an important gap, given that few research document the systematic process of adaptation of a health intervention.

The usability testing findings suggest it will be feasible to implement the intervention protocols, the intervention materials and contents are acceptable for the target audience and will support the objectives of the intervention. In addition, we garnered information that would help to enhance future recruitment and retention for a larger study. Health is Wealth usability testing showed that the intervention was well received. A brief educational session has the potential to address multiple barriers to cervical cancer screening using HPV self-sampling and increase knowledge of cervical cancer risks. Our next step is to implement and evaluate the preliminary effectiveness of the intervention with AA and SAI women in the community. If the intervention is implemented successfully, it will provide preliminary efficacy and feasibility data for a larger randomized controlled clinical trial for minority and medically underserved women. It will begin to fill an important literature gap regarding the feasibility of using peer educators and providing HPV self-sampling for cervical cancer screening promotion among AA and SAI women.

Progress is continuously being made to reduce cervical cancer burden and promote screening uptake. Future research should consider and compare the acceptability of other emerging HPV self-sampling methods such as the use of urine as a liquid biopsy for HPV DNA testing to offer a more accessible screening option among women who may consider cervicovaginal HPV self-sampling option as invasive [37].

### 5.1. Limitations

Despite the systematic processes used in the study, limitations exist. The generalizability of the findings is limited by convenience sampling and the small sample sizes. Despite these limitations, this study provided strong support for the adaptation and content of a cervical health intervention to increase cervical cancer screening.

### 5.2. Implication for Practice and Conclusions

The creation of an intervention without relying on any previous work may not be necessary if there is a tested evidence-based intervention that can be adapted to the needs of the target population. The adapted intervention is short, delivered via a PowerPoint presentation, and has the potential to be delivered to groups in community sites or clinic waiting rooms at low cost increasing its sustainability [38]. Documentation of the adaptation process ensures transparency and can inform the reproducibility of the intervention protocol. We demonstrated that an evidence-based intervention for Pap screening can be adapted for HPV-self-sampling promotion with target community input. It is important to engage with the target population during the development phases to ensure that the education content is relevant, appropriate, and feasible. The involvement and collaboration with communities and organizations in program design, adaptation, modifications, effective recruitment techniques, full-scale implementation, program evaluation, and interpretation of the results are relevant in improving outcomes. In conclusion, culturally and contextually adapted interventions can improve participants’ acceptability and engagement with the intervention leading to improved outcomes.

## Figures and Tables

**Table 1:** Summary of focus-group discussions to adapt education intervention for AA and SAI: emergent themes and exemplary quotes.

Themes	Concepts	Exemplar Quotes
1. Empowerment	Kit-use demonstration	“Some just really don’t like Pap smears or any type of you know, gynecologist visit. So the option along with convenience of doing it at home, but also, I’m in control of, you know, inserting this into my body and not a stranger or not my gynecologist. But you need to show me how to do it properly, I don’t want to hurt myself.” [Participant 1, grp 3, AA].
	Accessibility	“If it’s free of charge, I’m sure if people know the benefits, and they would definitely be willing to try it. And then if it’s something that’s something like just get mailed, some and mailed back, then that makes it like easy, I know like scheduling doctor’s appointments is like another thing, people like black women are probably going to be working during the week and it’s like when do you have time to even schedule these appointments?” [Participant 1, grp 4, SAI].
	Risk reduction, transmission, and prevention	“I will want to be advised on how to live a better life and how not to spread it around or get other people contaminated and to know how it spreads from one person to another. So that I cannot get other people infected, that if you don’t know out of ignorance, you might be living a very risky life in getting it across to a lot of people. I mean, if you’re positive, you really have to get some counseling to know how not to transmit it to other people. So, it’s still important to know whether you’re positive or negative, very important.” [Participant 2, grp 6, AA].
2. Comprehensive cervical health education	Self-sampling collection instruction	“I feel like if there’s instructions, I can go off and do it. And if I don’t do it right the first time then I’ll definitely go on to the second time around. But for convenience sake I would rather want to do this.” [Participant 1, grp 2, AA].
		“I don’t know may be there is more ways of passing the message across that is not harmful, it has benefits. On the Long run it is for your own good and not for any other things. Maybe just creating a lot of awareness about it.” [Participant 1, grp 1, SAI].
	Emphasize benefits of test	“I was thinking it could eliminate any stigma that, you know, maybe a black woman may have when it comes to needing checkups. Another thing that I see, like an advantage is that, you know, you can do it at your own time. So you won’t have make an appointment. And another thing here is it’s free. And that’s always good things. And if the accuracy is similar to you know what they do in the clinic, then that’s even better.” [Participant 3, grp 2, AA].
	Next steps following results	“Other information should include follow-up plans, frequency of test, hospital or clinic visit, if necessary, interval when tests should be done if negative.” [Participant 4, grp 4, SAI].
3. Confidentiality	Protect my identity	So for me, I think it’s just easy, you know, and maybe they would label it, I’m sure there’s a place where you put your name and all that, before you get the kit, but then everything is like with your ID and all that on the tube where they are gonna use to send it back. So all you need to do is this is already recorded, they are not gonna put any names on it, just numbers, and this is just to promote confidentiality.” [Participant 2, grp 6, SAI].
	Privacy of results	“I want to do it myself. My privacy matters. If I want to do to myself, I know how to be careful with myself.” [Participant 5, grp 6, SAI].
		“This depends on the result. It could be disclosed online through a passworded log in app but a positive result cannot be done this way. The client might have questions.” [Participant 2, grp 4, SAI].

**Table 2:** Usability testing ratings.

	Mean	SD
Information Acceptability		
The information was easy to understand	4.57	0.535
The information was credible	4.71	0.488
The information was important	4.57	0.535
The information was accurate	4.71	0.488
The information was relevant to me	4.71	0.488
Overall, I found the information helpful	4.71	0.488
Presentation Relevance		
The presentation addressed information I wanted to know about cervical cancer screening	4.57	0.535
The information improved my abilities to learn more about cervical cancer prevention	4.43	0.787
The presentation addresses strategies to improve preventive methods and behaviors	4.57	0.535
The presentation has the right information to promote cervical cancer screening	4.57	0.535
The information addressed any concern that I was having about cervical cancer screening	4.43	0.787
The presentation length is just right	4.71	0.488
The design of the PowerPoint is appealing	4.00	0.000
The amount of information presented is easy to get through	4.43	0.535
The Graphics/captions in the presentation are relevant for Black women	4.71	0.488
Usefulness of HPV Self-sampling Instruction		
The information in the kit presented the HPV self-sampling instruction clearly	4.29	0.756
The instruction is easy to understand and engaging	4.14	0.690
The video was very informative	4.29	0.756

**Table 3:** Original and adapted components of the intervention.

Intervention Component	Original	Adaptation	Adaptation Details
Population	Korean American women.	African American (AA) and sub-Saharan African Immigrant (SAI) women.	Culturally tailored for use with AA and SAI women. Slides included representative pictures of AA and SAI women.
Setting and recruitment	Community	Community-based Recruitment from community settings (churches, community centers, beauty supply stores), social media.	Recruitment settings expanded to reach AA and SAI women.
Eligibility	Women out of compliance with Pap screening. Women >21 years.	Women at average or high risk for cervical cancer. Women >30 years to 65 years	Revised to meet current recommendation for HPV testing based on the USPSTF recommendation.
Intervention contents	Education manual, resource sheet	(a) Education PowerPoint and brochure. (b) Video on HPV self-sampling process. (c) Information on available resources within the community. (d) HPV self-sampling kit provision	Revised for culturally appropriate language, graphics, images, and current statistics. Free screening resources in Lexington and Kentucky. Two case studies created. One case study focused on issues peculiar to AA and one case study focused on issues peculiar to SAI. HPV self-sampling provided to address access barrier.
Intervention time and session	2-h small group session	1 h small group session	Revised based on PowerPoint delivery time and feedback from Usability testing.
Intervention delivery	In-person delivery by trained navigator	Virtual delivery over Zoom by a trained peer educator. HPV kits mailed or hand delivered to participants.	Peer educator training revised to address issues specific to the AA and SAI women. Intervention script created. SAI peer educators deliver intervention for SAI women. AA peer educators deliver intervention for AA women.
Education content	2 h small-group session. Session addressed cervical cancer risk factors, screening, and potential barriers to screening. Patient navigator services. Reminder letter to schedule a Pap test.	(a) Awareness of cervical screening. (b) 1 h small-group session. (c) The session had 7 components which address cervical cancer risk factors, screening, and potential barriers to screening. Information on existing free and low-cost screening resources in the community. (d) Training on HPV self-sampling collection. (e) Services to help with follow-up if positive HPV test.	(a) Revised to address issues specific to the AA and SAI women. (b) HPV self-sampling instructions included in the kit. (c) Revised to address barriers identified through information gathered from focus-group sessions and the literature. (d) Follow-up call at 1 month to discuss their experience using HPV self-sampling. (e) Women who test positive for HPV are offered assistance in scheduling an appointment for a Pap test and navigated to follow-up care within 1 month of receiving the result.
HPV test Result delivery			Results are provided via a passworded email to the participants.
Required for intervention. implementation	(a) The Gateway to Health slide presentation. (b) Intervention Curriculum. (c) Reminder Letter.	(a) The Health is Wealth PowerPoint slide presentation. (b) HPV kits. (c) One follow-up call.	Slides were updated with content on current screening guidelines, and potential barriers to screening among Black women. Script and open-ended questions for a one-month follow-up call.

## Data Availability

The data that support the findings of this study are available from the corresponding author, upon reasonable request.
